# Supplementary material for: Identification of novel genetic regions associated with resistance to European canker in apple
Source: BMC Plant Biol. 2022 Sep 21;22:452. doi: 10.1186/s12870-022-03833-0 (PMC9490996; doi:10.1186/s12870-022-03833-0)
Supplement: Supplementary file 1 — Additional file 1: Supplementary Fig. 1A-1H. Phenotypic distributions for each European canker phenotype by family. Supplementary Fig. 2. Best Linear Unbiased Estimates (BLUEs) of parents and standard varieties. Supplementary Fig. 3. Scree plot from the principal component analysis of European canker phenotypes. Supplementary Fig. 4. Phenotypic distributions for progeny of ‘Golden Delicious’ x ‘M9’. [file 12870_2022_3833_MOESM1_ESM.pdf]

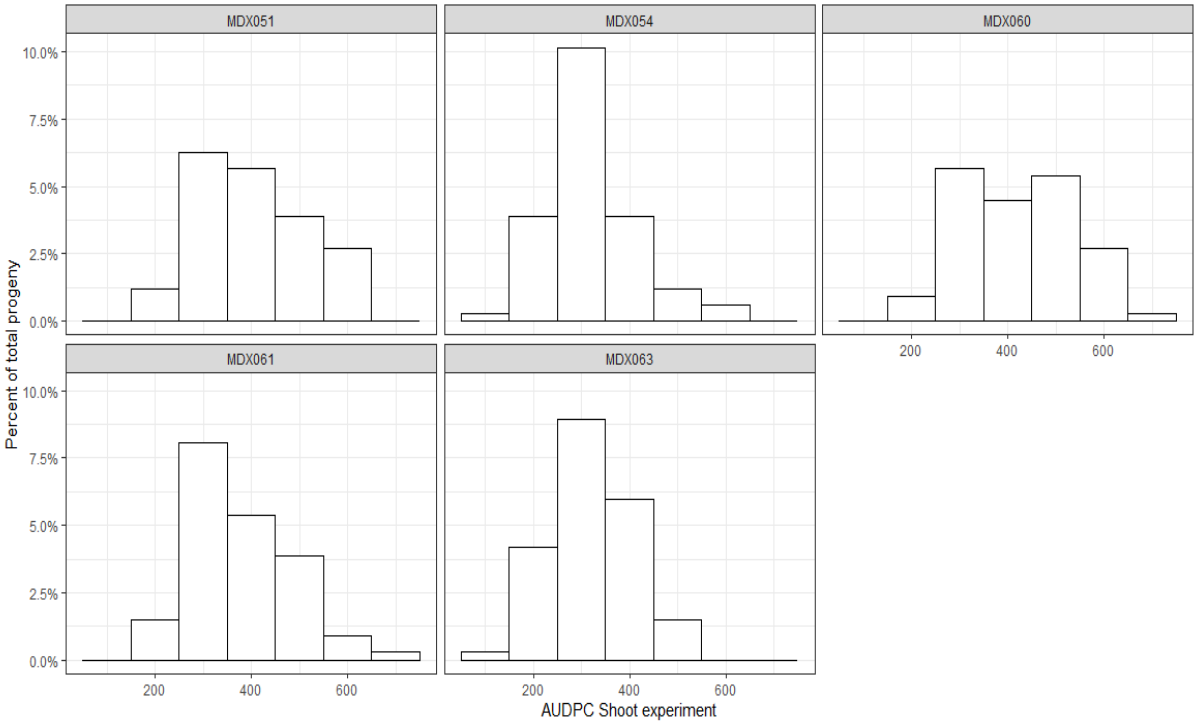

Supplementary figure 1A. Phenotypic distribution of Area Under Disease Progression Curve (AUDPC) in response to European canker in the shoot experiment. Distribution shown by family.

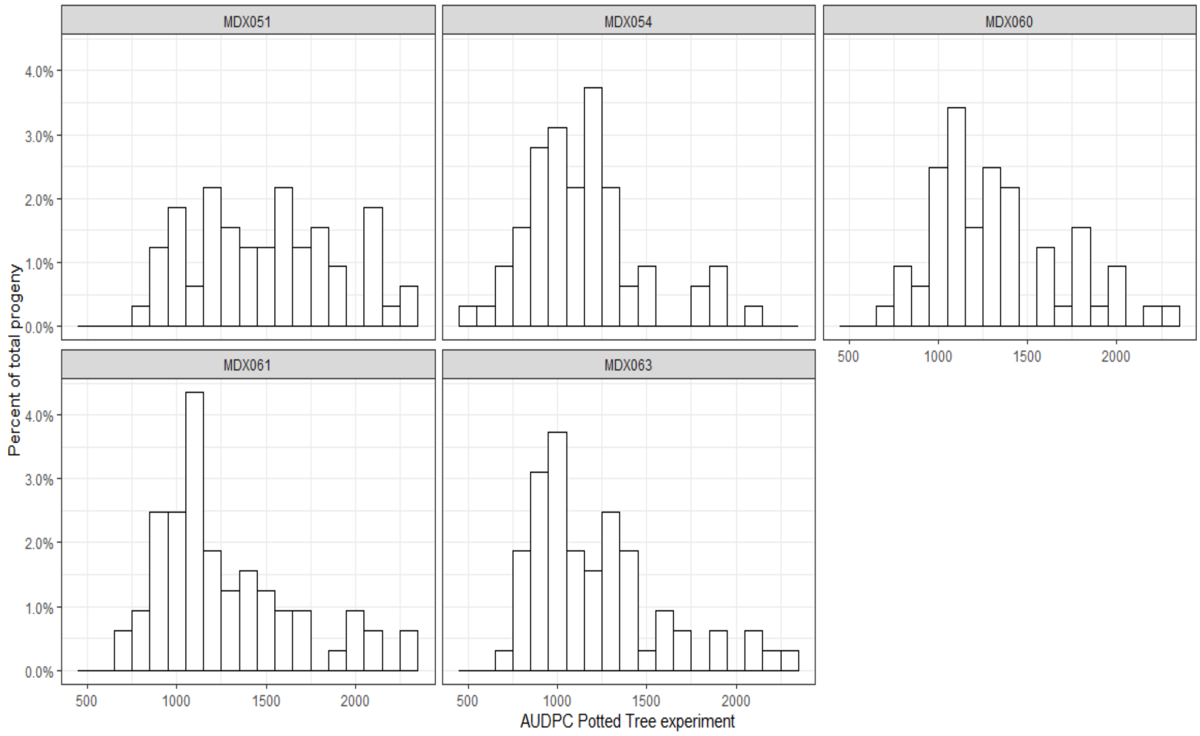

Supplementary figure 1B. Phenotypic distributions of Area Under Disease Progression Curve (AUDPC) in response to European canker in the potted tree experiment. Distribution shown by family.

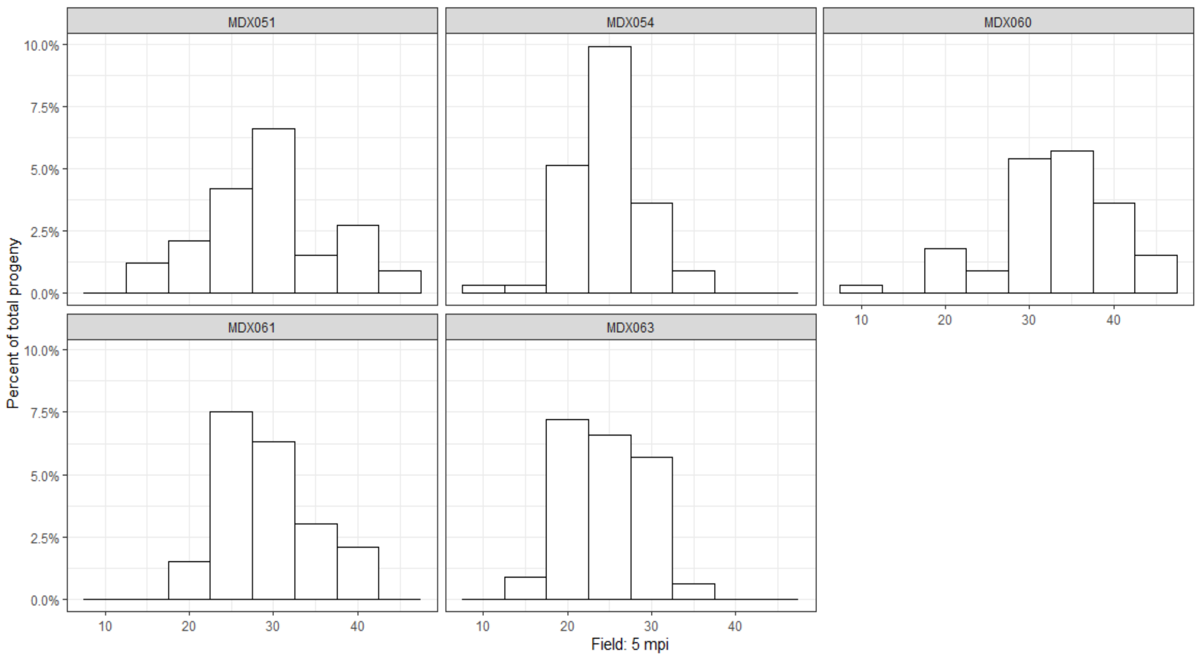

Supplementary figure 1C. Phenotypic distributions of lesion length (in mm) after infection with European canker in Field 5 months post inoculation. Distribution shown by family.

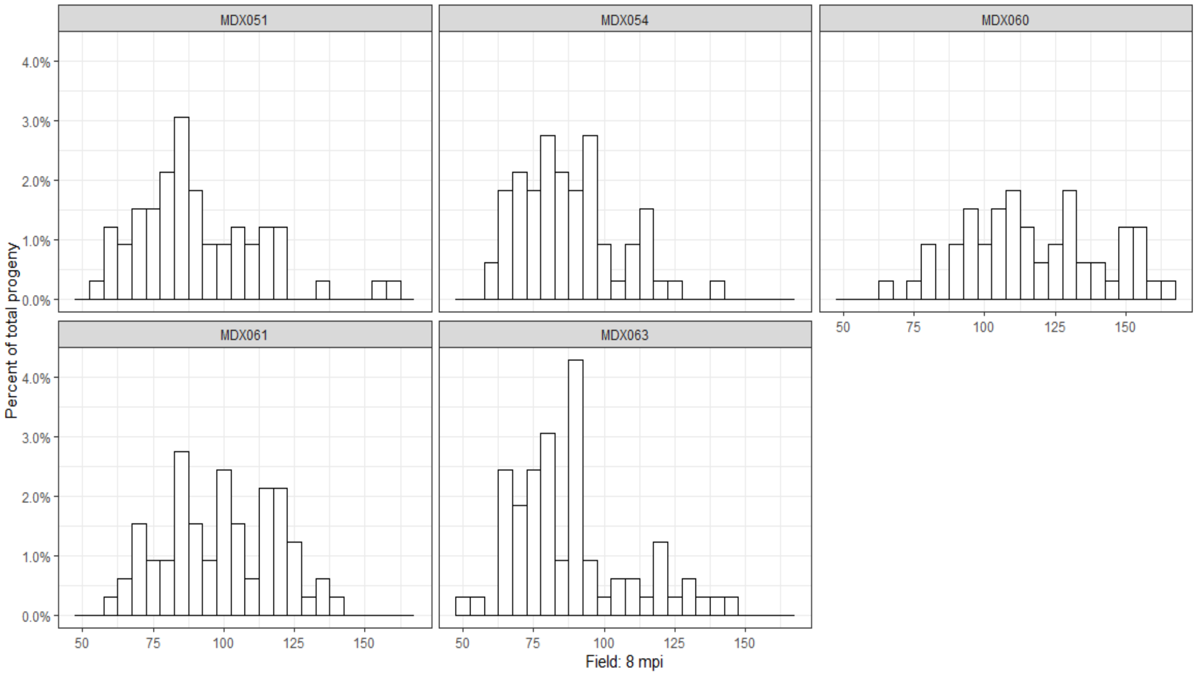

Supplementary figure 1D. Phenotypic distributions of lesion length (in mm) after infection with European canker in Field 8 months post inoculation. Distribution shown by family.

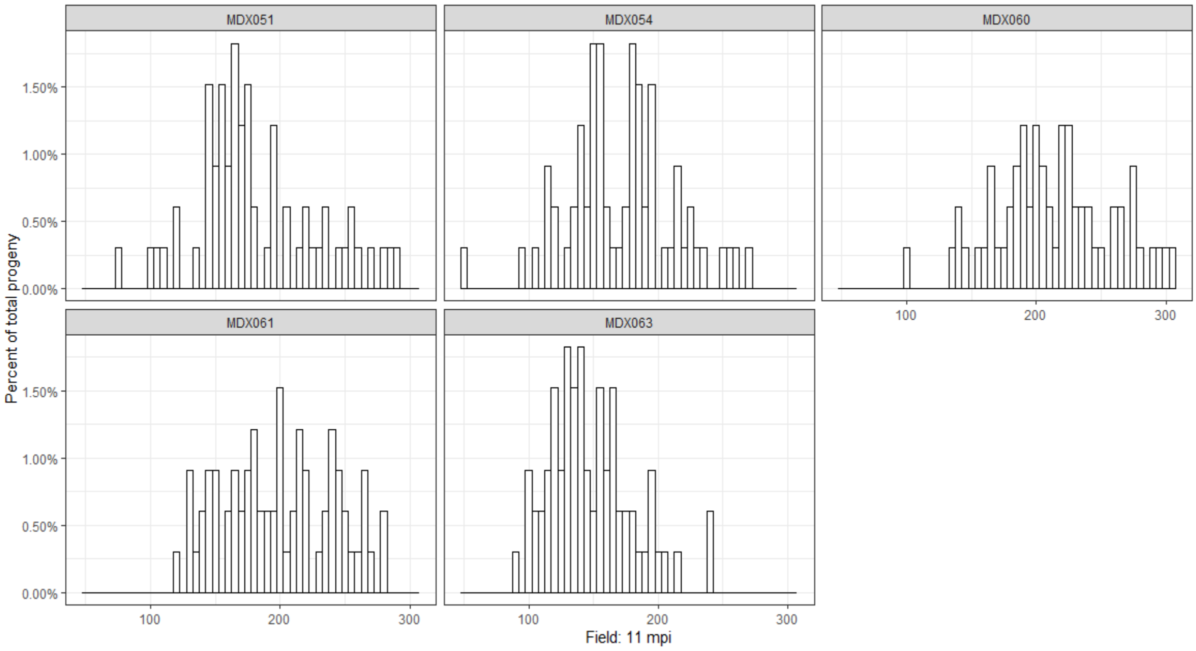

Supplementary figure 1E. Phenotypic distributions of lesion length (in mm) after infection with European canker in Field 11 months post inoculation. Distribution shown by family.

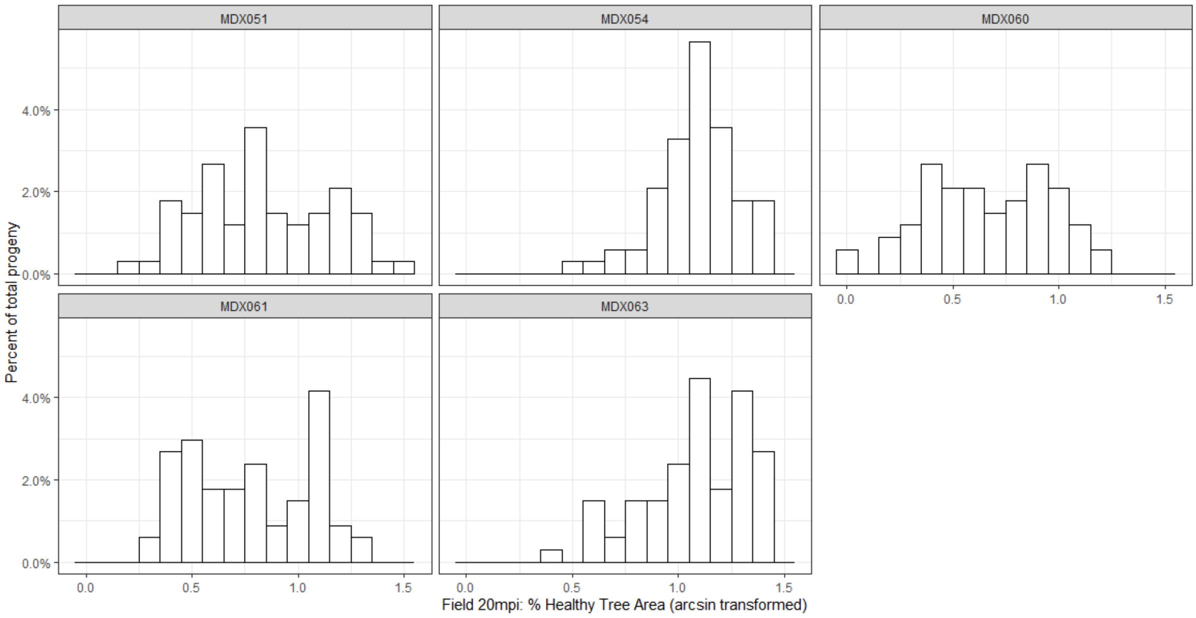

Supplementary figure 1F. Phenotypic distributions of arcsin transformed data for susceptibility to European canker in Field 20 months post inoculation: % Healthy Tree Area. Distribution shown by family.

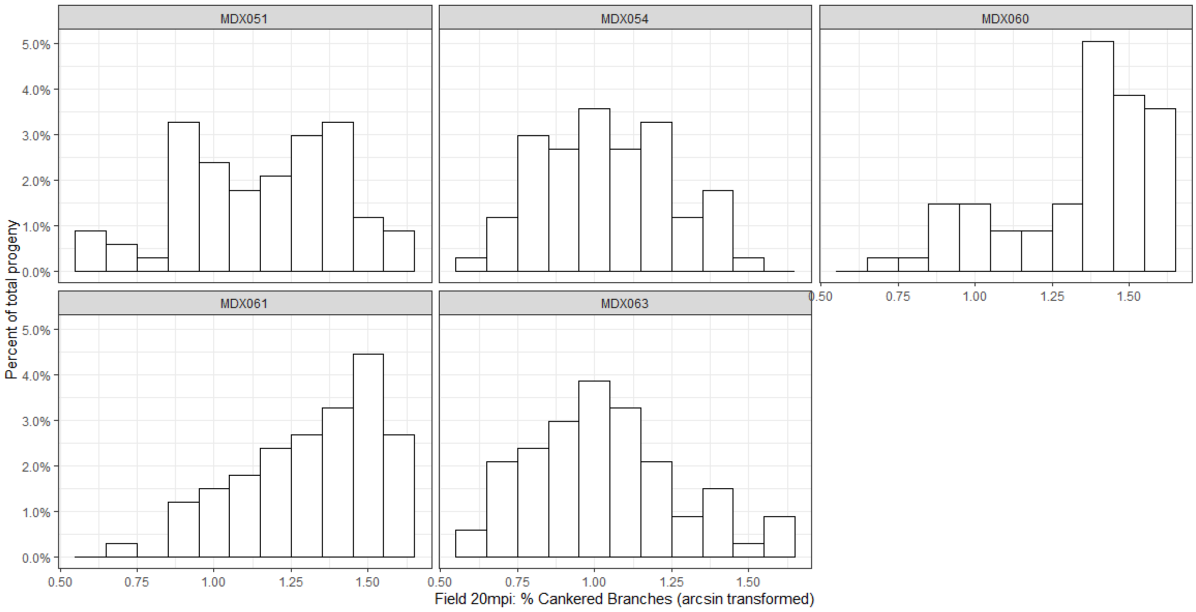

Supplementary figure 1G. Phenotypic distributions of arcsin transformed data for susceptibility to European canker in Field 20 months post inoculation: % Cankered Branches. Distribution shown by family.

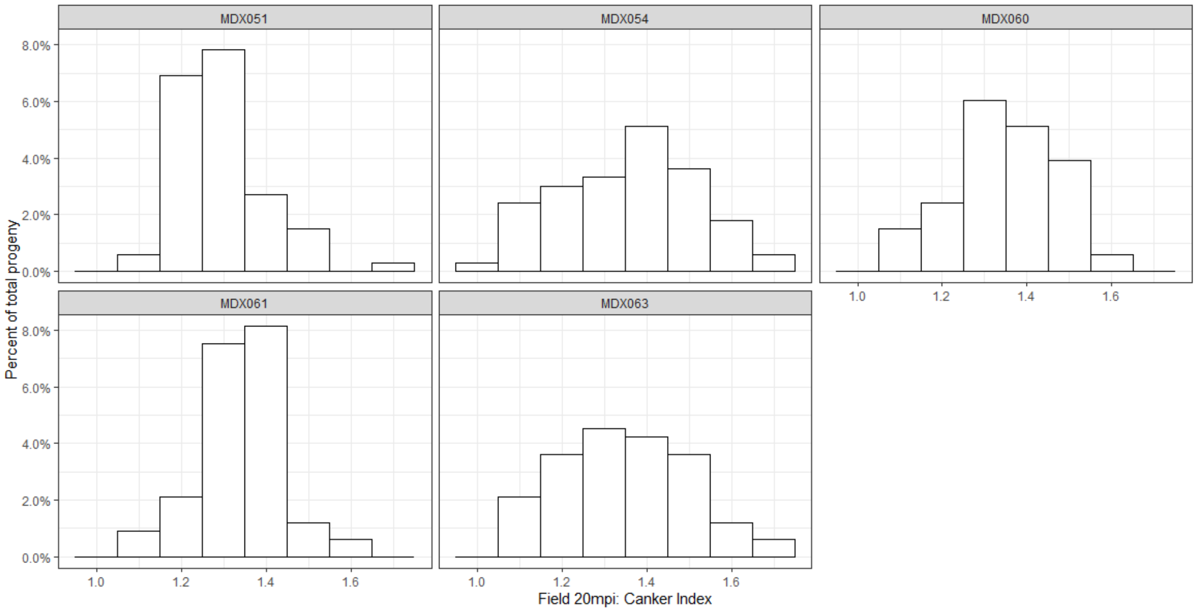

Supplementary figure 1H. Phenotypic distributions data for susceptibility to European canker in Field 20 months post inoculation: Canker Index. Distribution shown by family.

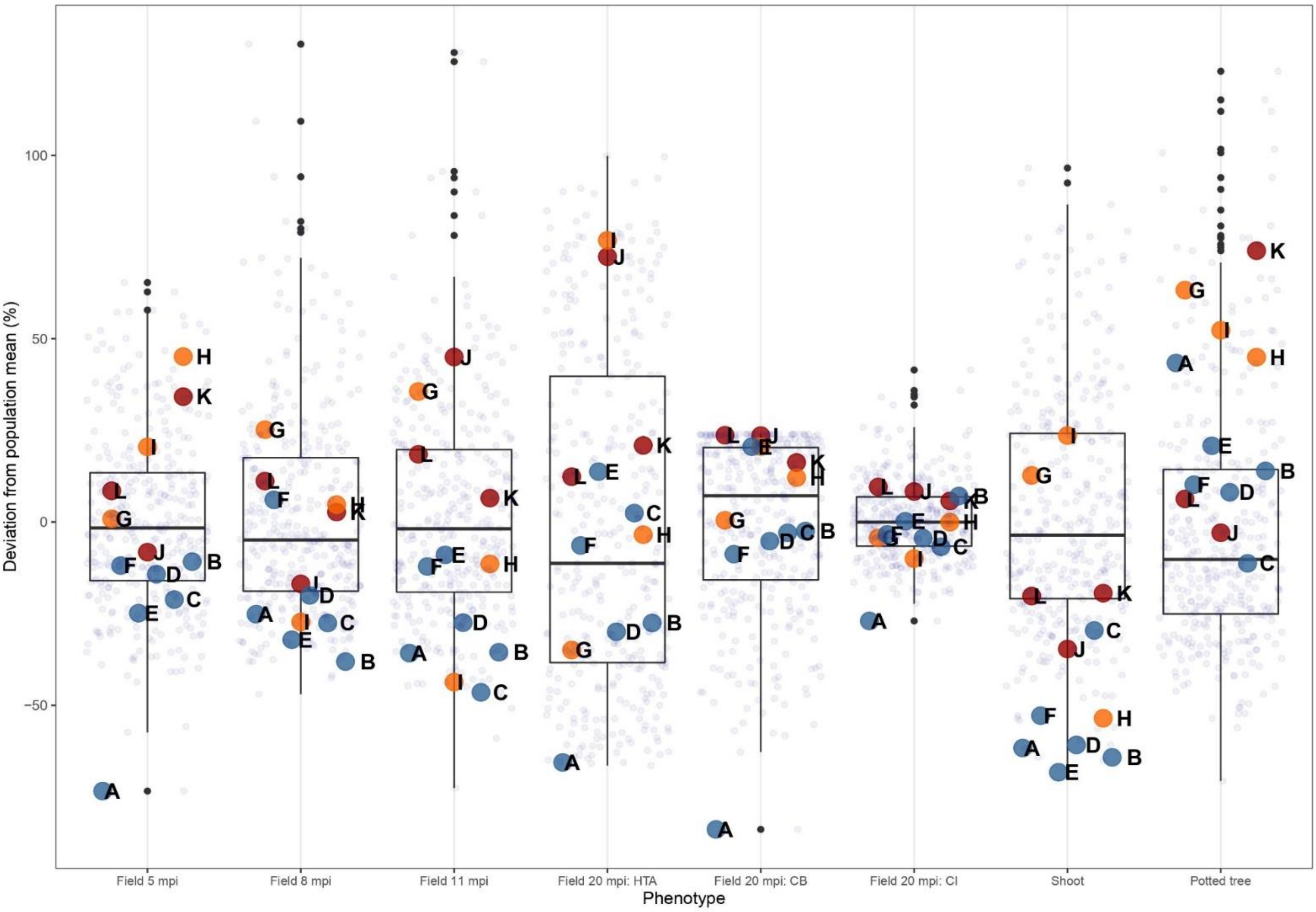

Supplementary figure 2. Best Linear Unbiased Estimates (BLUEs) of the multiparental population for each European canker phenotype. The data are shown as percent deviation from population mean, without the removal of outliers. Parents and standards are highlighted in colour, where the codes A-L refer to **(A)** seedling of *Malus robusta,* **(B)** Santana, **(C)** Jonathan, **(D)** Golden Delicious, **(E)** Elstar, **(F)** EM-Selection-1, **(G)** Aroma, **(H),** EM-Selection-2, **(I)** Cox Orange Pippin, **(J)** EM-Selection-4, **(K)** Gala, **(L)** EM-Selection-4. The inverse of %HTA is shown.

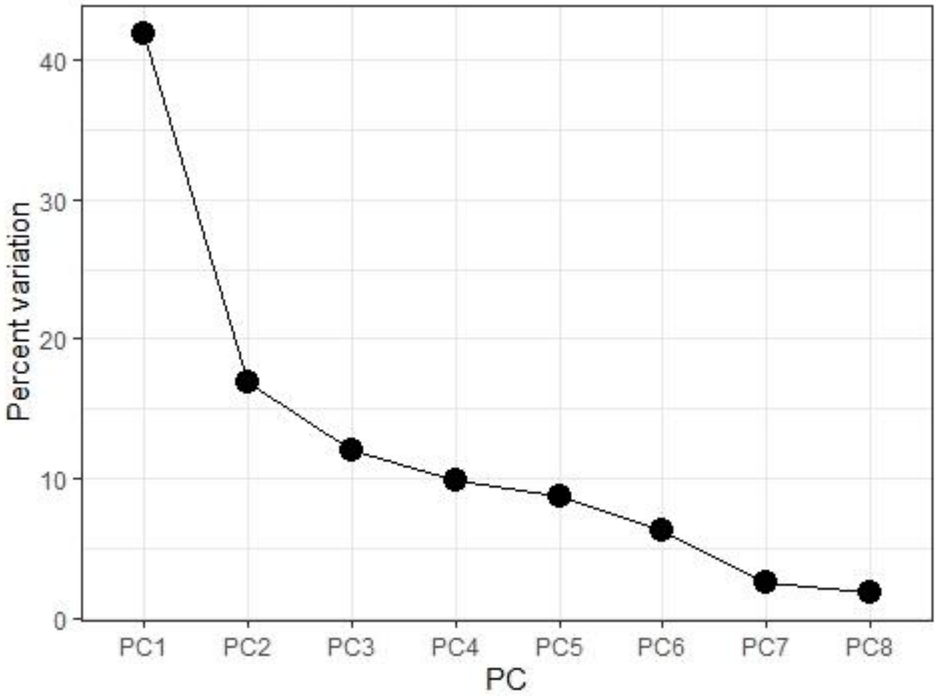

Supplementary figure 3. Scree plot from the principal component analysis (PCA) of the European canker phenotype data.

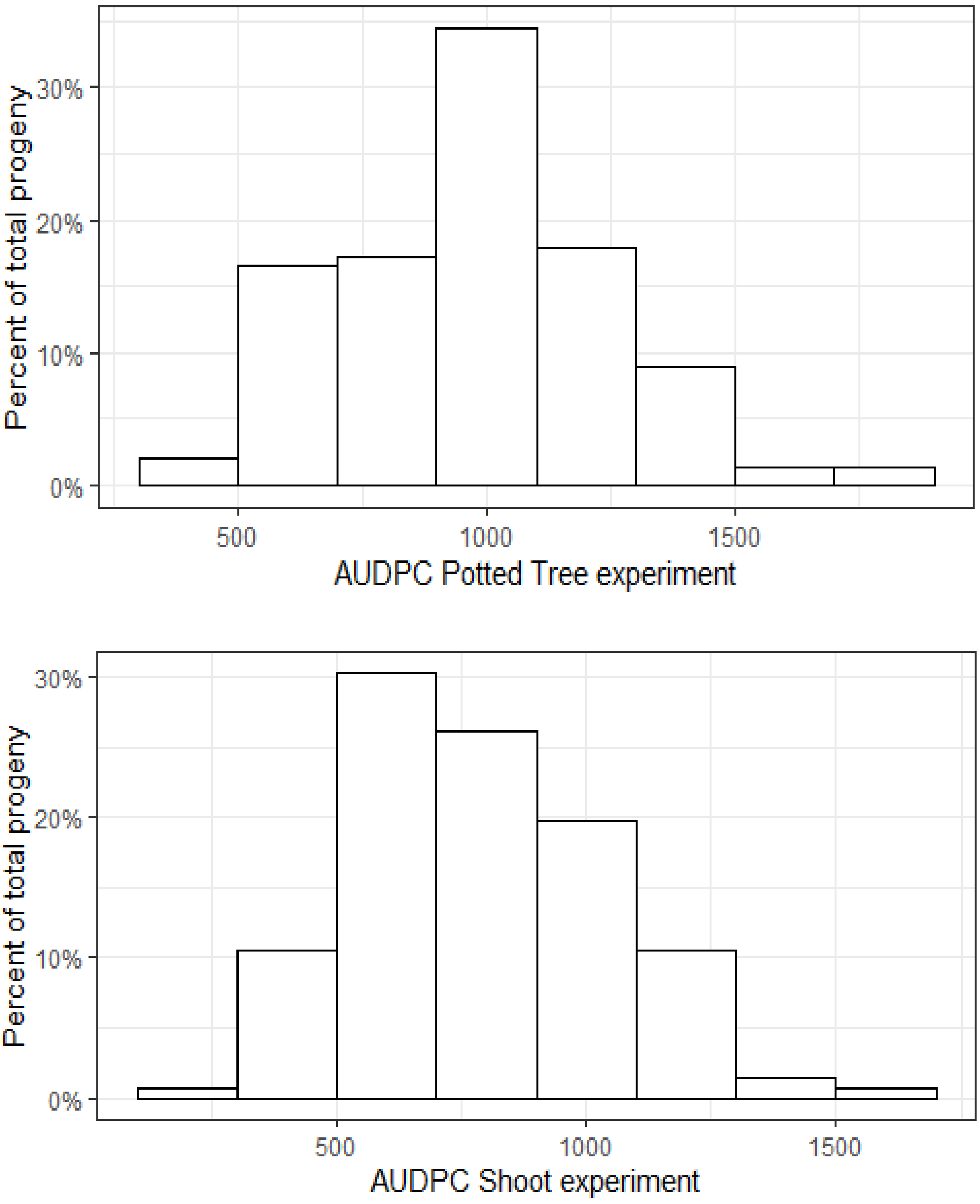

Supplementary figure 4. Phenotypic distributions for progeny of ‘Golden Delicious’ x ‘M9’ for susceptibility to European canker in a potted tree and a shoot experiment.
